# Two cases of pneumococcal spondylitis in the same household: a case report

**DOI:** 10.1186/s12879-018-3588-5

**Published:** 2018-12-17

**Authors:** Ken Goda, Tsuneaki Kenzaka, Bin Chang, Hozuka Akita

**Affiliations:** 1 Department of Internal Medicine, Hyogo Prefectural Kaibara Hospital, 5208-1, Kaibara, Kaibara-cho, Tanba, Hyogo 669-3395 Japan; 2 Division of Community Medicine and Career Development, Kobe University Graduate School of Medicine, 2-1-5, Arata-cho, Hyogo-ku, Kobe, Hyogo 652-0032 Japan; 3 Department of Bacteriology I, National Institute of Infectious Diseases, Tokyo, Japan

**Keywords:** Pneumococcal infection, Fever, Spondylitis, Bacteremia

## Abstract

**Background:**

Pneumococci normally reside in the nasopharynx, and when individuals are in close contact with each other such as in a community or a family setting, it is transmitted from carriers and sometimes results in pneumonia.

**Case presentation:**

Case 1: The patient was a 55-year-old woman who visited the hospital complaining of fever and headache. Lumbar pain occurred on hospital day 2, and purulent spondylitis was diagnosed using lumbar MRI. Blood culture results were positive for pneumococcus.

Case 2: The patient was a 60-year-old male, and the husband of the woman in the Case 1. Fever and lumbar pain occurred on the same day similar to Case 1. Inpatient treatment was provided for pneumococcal bacteremia. Although no abnormalities were observed on the lumbar MRI scan taken on hospital day 2, purulent spondylitis was diagnosed by an MRI taken on hospital day 9. Both patients received appropriate antimicrobial treatment. When bacterial strain analysis was performed on samples from Cases 1 and 2, we noted that the capsule serotype was 12F, the drug sensitivity was similar, and the sequence typing matched completely, indicating that the causative bacteria for both cases were identical.

**Conclusions:**

Pneumococcal bacteremia and purulent spondylitis can occur in different members of a family simultaneously. Pneumococcal infection can transmit between two close family members; hence, whenever a close family member of an individual who has already been infected with pneumococcal infection, develops fever, the possibility of transmission must be considered.

## Background

Pneumococci normally reside in the nasopharynx of an infant and can cause pneumonia, sepsis, and meningitis in susceptible individuals [1]. Pneumococcal infection is known to be transmitted in the carrier state not only in infants but also in adults in community and family settings where people live in close proximity to each other [2, 3]. Pneumococcal pneumonia infection is reportedly transmitted among persons living in close contact with each other, such as those within a family [4] or, as a rare occurrence, among nursing home residents [1].

## Case presentation

### Case 1

**Fig. 1:**
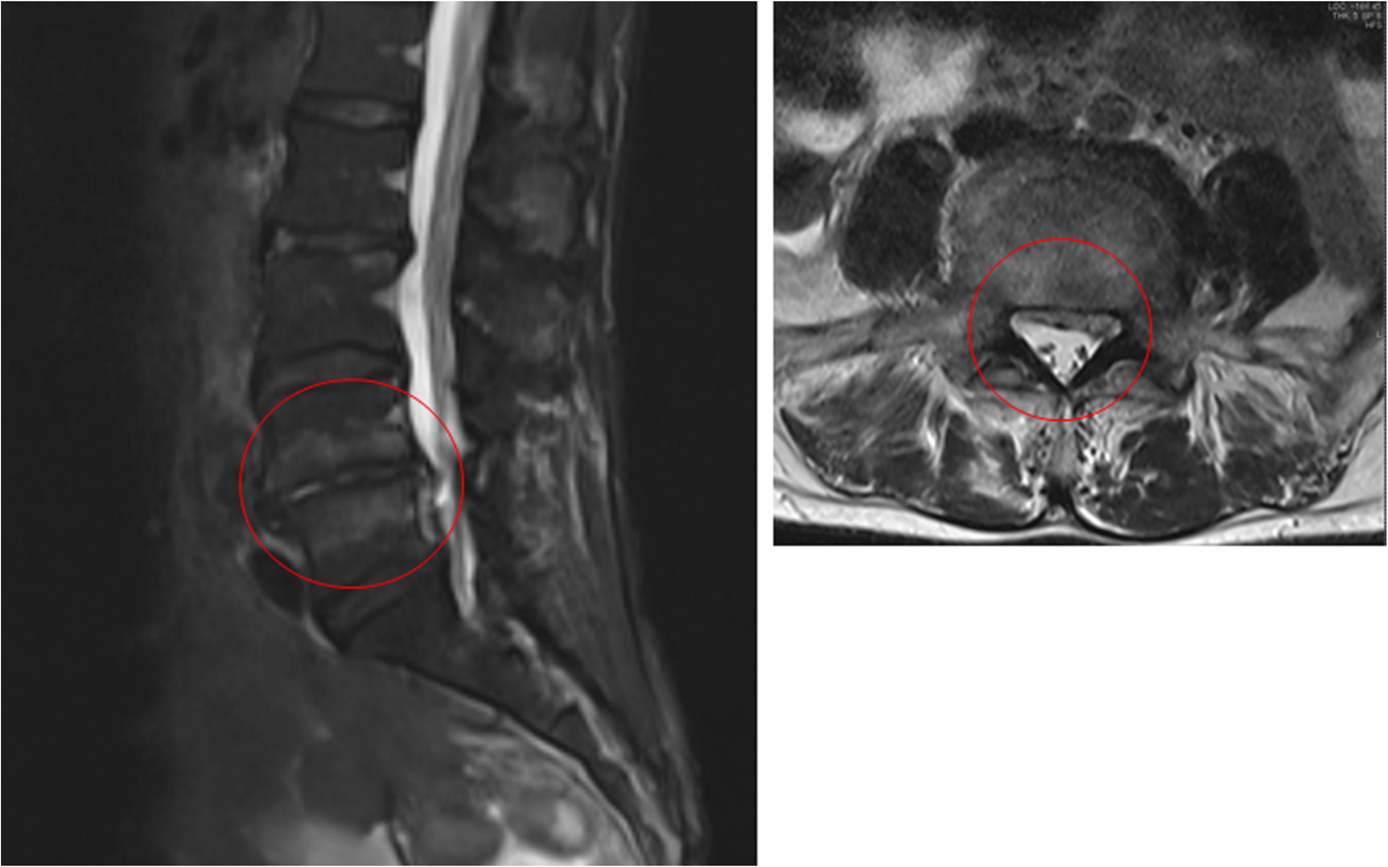
Lumbar MRI-short-tau inversion recovery (STIR) image (hospital day 3). An STIR image shows mildly hyperintense signal (red circle) in the vertebral body endplate of lumbar segments 4 and 5, which dips down on the posterior side of the 5th lumbar vertebra

The patient was a 55-year-old woman who visited the emergency room in the morning complaining of headache and fever. She was able to perform her daily activities independently and lived with her infant grandchild. She had no medical history of pneumonia and no history of pneumococcal vaccination. She received treatment for diabetes mellitus. The rapid influenza test result was negative, but blood samples were taken for culture test after which the patient was sent home with supportive therapy. However, because the headache continued to worsen, she returned to the emergency room later that evening and was hospitalized. The results of the blood test taken at this stage are presented in Table 1. Gram-positive cocci were detected in the blood culture taken during the initial visit, and treatment was initiated with 2 g ceftriaxone every 24 h combined with 1 g vancomycin every 12 h. Lumbago occurred on hospital day 2. A plain lumbar T1-weighted MRI scan showed a low signal intensity in the vertebral body endplate of the 4th and 5th lumbar vertebrae, and a short-tau inversion recovery image showed a mildly hyperintense signal in the region dipping below the posterior side of the 5th lumbar vertebra (Fig. 1); consequently, purulent spondylitis and epidural abscess were diagnosed. The results of other tests such as the spinal fluid test, head MRI, thoracoabdominal contrast CT, and transesophageal echocardiography did not indicate infection. Pneumococcus was detected on blood culture, and the antimicrobial drugs were changed to 2 g ampicillin (ABPC) every 6 h from hospital day 3. The blood culture result on hospital day 3 was negative. To normalize the white blood cell count, CRP level, and blood sedimentation rate and to improve CT findings, antimicrobial drug treatment was administered for a total of 9 weeks.

**Table 1:** Laboratory data of Case 1 on admission

Parameter	Recorded value	Standard value
White blood cell count	8.27 × 10^9^/L	4.50–7.50 × 10^9^/μL
Neutrophils	91.5%	
Lymphocytes	6.9%	
Hemoglobin	12.7 g/dL	11.3–15.2 g/dL
Hematocrit	33.0%	36–45%
Platelets	141 × 10^9^/L	130–350 × 10^9^/L
C-reactive protein	10.1 mg/dL	≤0.60 mg/dL
Total protein	7.0 g/dL	6.9–8.4 g/dL
Albumin	3.8 g/dL	3.9–5.1 g/dL
Aspartate aminotransferase	25 U/L	11–30 U/L
Alanine aminotransferase	20 U/L	4–30 U/L
Lactate dehydrogenase	258 U/L	109–216 U/L
Creatine phosphokinase	119 U/L	40–150 U/L
Blood nitrogen urea	12.8 mg/dL	8–20 mg/dL
Creatinine	0.70 mg/dL	0.63–1.03 mg/dL
Sodium	133 mEq/L	136–148 mEq/L
Potassium	3.96 mEq/L	3.6–5.0 mEq/L
Glucose	215 mg/dL	70–109 mg/dL
Hemoglobin A1c	7.7%	≤6.5%

### Case 2

**Fig. 2:**
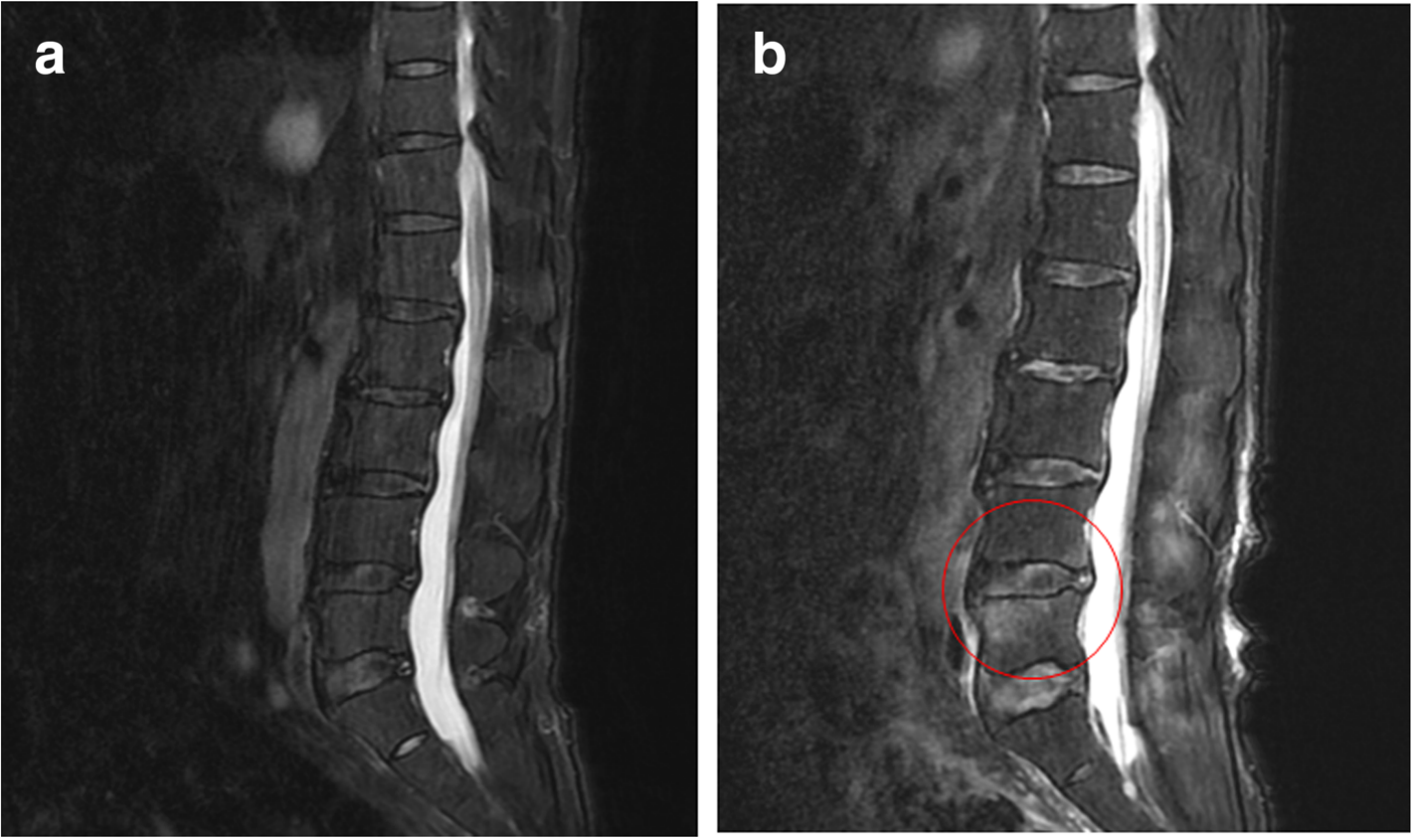
Lumbar MRI-STIR image. **a** No abnormalities on hospital day 2 were noted (**b**) A mildly hyperintense signal was observed at lumbar segments 4 and 5 on hospital day 9 (red circle)

The patient was a 60-year-old man who was the husband of the patient described above. He experienced lumbago on the same morning his wife was hospitalized. Fever and disturbance of consciousness occurred in the evening, and he visited the emergency room at another hospital. He had completed the course of neoadjuvant and adjuvant chemotherapy and radiation for glioblastoma 6 years ago, and was able to carry out his daily activities independently. He had no medical history of pneumonia or a history of pneumococcal vaccination. Although right lumbar pain was observed, the origin of fever was unknown; antimicrobial treatment was initiated with 4.5 g piperazine/tazobactam every 8 h. The results of the blood test taken at hospitalization are shown in Table 2. Pneumococcus was detected in his blood culture as well, and the regimen was changed to 2 g ABPC every 6 h. There were abnormal findings in the lumbar MRI scan taken on hospital day 2. Nonetheless, the results of the spinal fluid test, head MRI, thoracoabdominal contrast CT, transesophageal echocardiography, did not reveal presence of infection at other sites. Because lumbago persisted, MRI was performed again on hospital day 9; the consequent MRI results revealed purulent spondylitis (Fig. 2). The patient was transferred to our hospital on hospital day 9. To normalize white blood cell count, CRP level, and blood sedimentation rate and to improve CT findings, antimicrobial drug treatment was carried out for a total of 9 weeks.

**Table 2:** Laboratory data of Case 2 on admission

Parameter	Recorded value	Standard value
White blood cell count	8.75 × 10^9^/L	4.50–7.50 × 10^9^/μL
Neutrophils	82.4%	
Lymphocytes	13.6%	
Hemoglobin	16.4 g/dL	11.3–15.2 g/dL
Hematocrit	49.5%	36–45%
Platelets	81 × 10^9^/L	130–350 × 10^9^/L
C-reactive protein	25.0 mg/dL	≤0.60 mg/dL
Total protein	6.3 g/dL	6.9–8.4 g/dL
Albumin	3.7 g/dL	3.9–5.1 g/dL
Aspartate aminotransferase	182 U/L	11–30 U/L
Alanine aminotransferase	102 U/L	4–30 U/L
Lactate dehydrogenase	396 U/L	109–216 U/L
Creatine phosphokinase	360 U/L	40–150 U/L
Blood nitrogen urea	45.0 mg/dL	8–20 mg/dL
Creatinine	1.02 mg/dL	0.63–1.03 mg/dL
Sodium	142 mEq/L	136–148 mEq/L
Potassium	3.9 mEq/L	3.6–5.0 mEq/L
Glucose	122 mg/dL	70–109 mg/dL
Hemoglobin A1c	5.7%	≤6.5%

Both patients tolerated the antimicrobial treatment well, and recovered completely. A 13-valent pneumococcal conjugate vaccine was administered initially, and a 23-valent pneumococcal polysaccharide vaccine was administered 1 year later in both patients. More than 1 year has elapsed since the completion of treatment, and there has been no recurrence.

In the bacterial strain analysis (Table 3) performed on the samples from both patients, the capsule serotype was 12F identified by the capsule quelling reaction using rabbit antisera (Statens Serum Institute, Copenhagen, Denmark). Drug sensitivity test was performed using a dry plate Eiken (Eiken Chemical Co., Tokyo, Japan), and was performed in accordance with CLSI M100-S-18 (hemosupplemented Mueller–Hinton broth, 22-h culture). The drug sensitivity in both strains was similar. As the next step, we performed a sequence typing match using the defined genetic sequence of the pneumococci was determined (aroE, gdh, gki, recP, spi, xpt, ddl) according to the method described in http://spneumoniae.mlst.net/, and it was compared with the sequence information present in the existing databases. Both strains matched completely.

**Table 3:** Results of bacterial strain analysis

By serotype						Serotype					
Case 1						Type 12F					
Case 2						Type 12F					
Drug sensitivity (g/mL)											
Strain	PCG	ABPC	CTX	TBPM	PAPM	MEPM	CDTR	EM	CLDM	VCM	TFLX
Case 1	0.06	≤0.03	≤0.03	≤0.008	≤0.008	0.015	≤0.03	≥8	≥8	0.25	≤0.12
Case 2	0.06	≤0.03	0.06	≤0.008	≤0.008	0.015	≤0.03	≥8	≥8	0.25	≤0.12
Sequence typing											
Strain		ST	*aroE*	*Gdh*	*Gki*		*recP*	*spi*	*xpt*	*ddl*	
Case 1		4846	12	32	111		1	13	48	6	
Case 2		4846	12	32	111		1	13	48	6	

## Discussion and conclusions

Here, we report the case of pneumococcal bacteremia and purulent spondylitis occurring simultaneously in both a husband and wife. To the best of our knowledge, this is the first report in which pneumococcal bacteremia and purulent spondylitis have occurred simultaneously.

These cases revealed the following findings: 1) pneumococcal bacteremia and purulent spondylitis occurred in both patients; 2) when purulent spondylitis is suspected, imaging tests, if initially negative, should be repeated.

Pneumococcal infection is known to be transmitted in the carrier state among adults in community and family settings, where they are in close proximity with one another [2, 3]. Moreover, the transmission of pneumococcal pneumonia has been reported to occur within families [4, 5]^.^ In the present case, it was assumed that the infant living with the patients (i.e., the patients’ grandchild) was a carrier, and contact between them resulted in simultaneous infection; however, bacteriological tests were not performed for the infant for ethical and health insurance purposes.

In Japan, 14.2% of the pneumococci isolated from invasive pneumococcal infection between August 2006 and July 2007 were reported to be of serotype 12F [6]. In the present case, the capsule serotype of the airborne-transmitted pneumococci was of the highly invasive type 12F, which is considered to have led to the purulent spondylitis with bacteremia. Since the serotype of the pneumococcal strain that had led to the rare complication of spondylitis, was the same in both patients, we decided to explore virulence factors other than the serotype, which confirmed these bacteria were identical.

It has been reported that the imaging findings of purulent spondylitis might be delayed [7, 8]. Hence, if physical findings are repeatedly evaluated and purulent spondylitis is suspected from localized pain or other symptoms that gradually appear at the vertebral site, MRI must be reperformed [7]. Persistent lumbago was observed in the present cases as well, and performing tests repeatedly when purulent spondylitis was suspected proved to be useful for diagnosis.

The Infectious Diseases Society of America (IDSA) guidelines [9] indicate a minimum of 6-week treatment for purulent spondylitis, since in cases where non-oral drug therapy is administered for less than 4 weeks, the relapse rate is reported to increase to 25% [10]. However, the period of antimicrobial drug administration for treating general infections is 14 days or less, and thus, early diagnosis is required; an appropriate treatment period will thereby contribute to a lower frequency of relapse.

In conclusion, a case of simultaneous occurrence of pneumococcal bacteremia and purulent spondylitis in a woman and her husband is described. Transmission of pneumococcal infection can occur between close family members; hence, when a close family member of an individual with confirmed pneumococcal infection presents with fever, the possibility of transmission of pneumococcal infection must be considered.

## Abbreviations

ABPC: Ampicillin
CLSI: Clinical and Laboratory Standards Institute
CRP: C-reactive protein
CT: Computed tomography
IDSA: Infectious Diseases Society of America
MRI: Magnetic resonance imaging
